# Tobacco Smoke Exposure in Children and Adolescents: Prevalence, Risk Factors and Co-Morbid Neuropsychiatric Conditions in a US Nationwide Study

**DOI:** 10.1192/j.eurpsy.2025.373

**Published:** 2025-08-26

**Authors:** M. Salehi, M. Saeidi, N. Kasulis, T. Barias, T. Kainth, S. Gunturu

**Affiliations:** 1 Department of Psychiatry, University of Minnesota School of Medicine, Minneapolis; 2 Department of Psychiatry, Bronx Care Health System; 3 Department of Psychiatry, Icahn School of Medicine at Mount Sinai, New York, United States

## Abstract

**Introduction:**

Tobacco smoke exposure(TSE) is a significant public health issue, children and adolescents, who are often involuntarily exposed through secondhand or thirdhand smoke.This exposure has been linked to a range of neuropsychiatric conditions and can negatively impact mental and physical well-being. In the U.S., TSE is prevalent among certain sociodemographic groups, including those with lower income and specific racial backgrounds.The goal of this study was to evaluate the prevalence of TSE and its association with neuropsychiatric comorbidities.

**Objectives:**

Assess TSE prevalence among U.S. children and adolescents

Examine sociodemographic factors influencing TSE

Analyze the link between TSE and neuropsychiatric conditions’ prevalence and severity

**Methods:**

This cross-sectional study used data from the 2020-2021 National Survey of Children’s Health (NSCH), a survey conducted by the U.S. Census Bureau.Parent-proxy responses were collected in English and Spanish through mail and web-based surveys.A total of 91,404 children aged 0-17 were included for the analysis of TSE prevalence, while 79,182 children aged 3-17 were analyzed for neuropsychiatric comorbidities.The primary measures were TSE, assessed through household smoking behavior, and the presence of neuropsychiatric conditions. Statistical analyses included t-tests, Chi-Square tests, and multivariate regression models to identify associations between TSE, socio-demographic factors, and neuropsychiatric comorbidities, providing adjusted odds ratios and confidence intervals. Statistical analyses were carried out using Stata version 17.

**Results:**

TSE was identified in 12.9% of the sample population. The likelihood of TSE was higher among males and adolescents aged 11-17 years. Children from lower-income households and American Indian/Alaska Native backgrounds had a greater risk of TSE. Among children exposed to tobacco smoke, 36.4% had at least one neuropsychiatric comorbidity, with anxiety (15.7%), ADHD (15.5%), and conduct problems (13.7%) being the most common. Females had lower odds of anxiety and autism spectrum disorder(ASD)compared to males, and Asian children exhibited lower odds of ADHD and other conditions. TSE was associated with increased severity of neuropsychiatric conditions. Figure 1.

**Image 1:**

**Figure:**
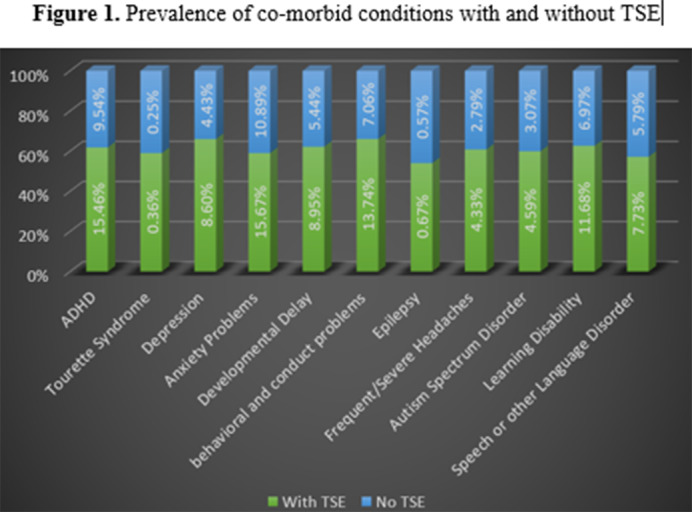

**Conclusions:**

TSE is a significant concern among U.S. children and adolescents, particularly affecting males and those from lower-income families. Exposure to tobacco smoke notably elevates the risk and severity of neuropsychiatric conditions, with ADHD, behavioral and conduct problems, and learning disabilities being the most common co-occurring issues. These findings highlight the importance of early screening and intervention for youth exposed to tobacco smoke. Addressing sociodemographic disparities in exposure and implementing prevention strategies could play a critical role in reducing these negative health outcomes.

**Disclosure of Interest:**

None Declared

